# Cardiac Implantable Electronic Device-Related Infective Endocarditis Caused by *Bacillus cereus*: A Case Report

**DOI:** 10.3390/jcm15010344

**Published:** 2026-01-02

**Authors:** Denis Swolana, Danuta Łoboda, Beata Sarecka-Hujar, Rafał Sznajder, Anna Szajerska-Kurasiewicz, Tadeusz Zębik, Krzysztof S. Gołba, Robert D. Wojtyczka

**Affiliations:** 1 Department of Microbiology, Faculty of Pharmaceutical Sciences in Sosnowiec, Medical University of Silesia in Katowice, 4 Jagiellońska Str., 41-200 Sosnowiec, Poland; dswolana@sum.edu.pl (D.S.); rwojtyczka@sum.edu.pl (R.D.W.); 2 Department of Electrocardiology, Upper-Silesian Medical Centre in Katowice, 45/47 Ziołowa Str., 40-635 Katowice, Poland; dana.loboda@gmail.com (D.Ł.); rafal.sznajder@gmail.com (R.S.); kgolba@sum.edu.pl (K.S.G.); 3 Department of Electrocardiology and Heart Failure, Faculty of Health Sciences in Katowice, Medical University of Silesia in Katowice, 45/47 Ziołowa Str., 40-635 Katowice, Poland; 4 Department of Basic Biomedical Science, Faculty of Pharmaceutical Sciences in Sosnowiec, Medical University of Silesia in Katowice, 8b Jedności Str., 41-200 Sosnowiec, Poland; 5 Department of Cardiology, Municipal Hospital in Gliwice, 29 Kosciuszki Str., 44-100 Gliwice, Poland; a.szajerska.kurasiewicz@gmail.com (A.S.-K.); t.zebik@szpital4.gliwice.pl (T.Z.)

**Keywords:** cardiac implantable electronic device, Gram-positive bacterial infections, infective endocarditis, pocket infection, case report

## Abstract

**Background:** Globalization, increased mobility, changes in dietary habits, and a growing number of immunocompromised patients have heightened exposure to rare or opportunistic pathogens. Here, we present a case of cardiac implantable electronic device-related infective endocarditis (CIED-IE) caused by *Bacillus cereus* bacteremia originating in the gastrointestinal tract. **Case presentation:** A 66-year-old female, who had a cardiac resynchronization pacemaker (CRT-P) implanted in 2017 due to second-degree atrioventricular block and left bundle branch block, had undergone device replacement due to battery depletion 4 months earlier and was scheduled for transvenous lead extraction (TLE) due to generator pocket infection. During the TLE procedure, transoesophageal echocardiography revealed vegetations on the leads and in the right atrium. Standard empirical therapy covering methicillin-resistant Staphylococci and Gram-negative bacteria was administered, including oritavancin and gentamicin. Surprisingly, intraoperative samples cultured *B. cereus*, a Gram-positive, spore-forming rod that usually causes food poisoning through contamination of rice and other starchy foods. *B. cereus* is generally resistant to β-lactam antibiotics except for carbapenems but is susceptible to glycopeptides. The oritavancin treatment was extended to four fractionated doses (1200, 800, 800, and 800 mg) administered at 7-day intervals. To eradicate bacteria in the gastrointestinal tract, oral vancomycin (125 mg 4 times a day) was added. After 4 weeks of effective antibiotic therapy, a CRT-P with a left bundle branch area pacing lead was reimplanted on the right subclavian area, with no recurrence of infection during the 3-month follow-up. **Clinical discussion:** In the patient, a diet high in rice and improper storage of rice dishes, together with habitual constipation, were identified as risk factors for the development of invasive *Bacillus cereus* infection. However, the long half-life lipoglycopeptide antibiotic, oritavancin, administered weekly, proved effective in treating CIED-IE. **Conclusions:** Infection with rare or opportunistic microorganisms may require extended microbiological diagnostics and non-standard antibiotic therapy; therefore, the medical history should consider risk factors for such infections.

## 1. Introduction

Infections caused by atypical or opportunistic pathogens are increasingly common today, driven by the growing number of immunocompromised patients [1]. It may naturally result from an aging population and the increasing proportion of individuals with naturally weakened immune systems [2]. The increased lifespan of patients with chronic diseases and the development of immunosuppressive or immunomodulatory therapies (e.g., oncology, hematology) mean that more people remain at risk of infection with invasive opportunistic pathogens [3].

Additionally, factors related to globalization, mobility, and changes in dietary behavior increase exposure to rare or previously unheard-of pathogens [4,5]. Changes in dietary habits, including the consumption of prepared, repeatedly heated, or stored foods, also increase the risk of transmission of opportunistic bacteria by contaminated food, which may be important in susceptible patients [6].

Infective endocarditis is the most serious complication of cardiac implantable electronic devices (CIEDs). In 40% of cases, it develops as a result of infection of the generator pocket, which spreads along the intravascular segments of the leads to the right atrium and ventricular endocardium. In the remaining cases, the blood-origin CIED infection occurs from a distant inflammatory site [7]. The most common etiologic agents of CIED infections are *Staphylococcus aureus* and coagulase-negative Staphylococci, often multidrug-resistant [8,9]. Less common are other Gram-positive bacteria, such as *Streptococcus* spp., *Enterococcus* spp., *Cutibacterium* spp., and *Corynebacterium*, or Gram-negative bacilli. CIED-related infections with other pathogens are sporadic and described as casuistic cases—they constitute no more than 1% of infections [8].

In this report, we present a case of CIED-related infective endocarditis (CIED-IE) caused by *Bacillus cereus* bacteremia originating in the gastrointestinal tract.

## 2. Case Presentation

A 66-year-old female patient underwent cardiac resynchronization therapy pacemaker (CRT-P) implantation in 2017 due to advanced atrioventricular and intraventricular conduction disturbances characterized by a PQ interval of 280 ms, paroxysmal second-degree atrioventricular block, and left bundle branch block with a QRS duration of up to 200 ms. Despite a normal left ventricular ejection fraction (55%), the predicted right ventricular pacing burden was high, and the electromechanical dyssynchrony was significant. Our academic center, the Department of Electrocardiology, Upper Silesian Medical Center in Katowice (Poland), has been conducting research on the prevention of pacing-induced cardiomyopathy for years, which explains the off-label use of CRT-P. The device required replacement due to battery depletion in April 2025. On 27 August 2025, the patient was urgently admitted to the hospital with suspected isolated infection of the generator pocket.

For several days before admission, the patient had noticed skin redness and tenderness in the generator pocket area. The patient reported no systemic symptoms such as fever or chills and had not experienced any infections elsewhere in recent weeks; the patient did not receive any outpatient antibiotic therapy. A year earlier, the patient experienced symptoms consistent with an intestinal infection accompanied by severe vomiting, which required an exploratory laparotomy to rule out intestinal obstruction. Furthermore, the patient reported habitual constipation. The basis of the patient’s diet was rice and groats. Any rice the patient did not eat was usually cooled to room temperature and refrigerated overnight. The following day, the rice was only lightly warmed.

**Figure 1:**
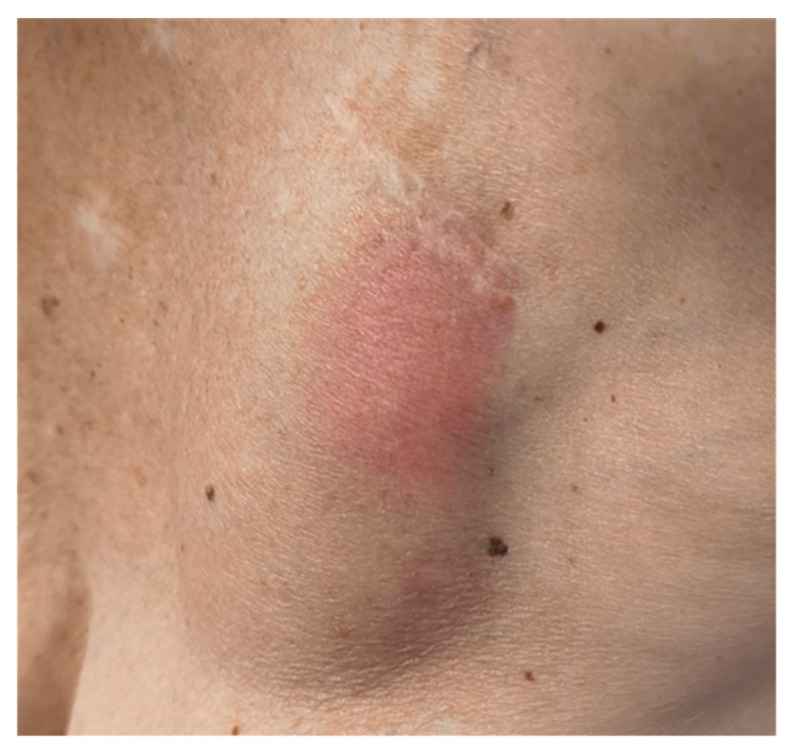
Erythema and swelling of the skin over the device in the left subclavian area.

Physical examination revealed pain, erythema, fluctuance, and swelling of the skin over the device (Figure 1).

**Figure 2:**
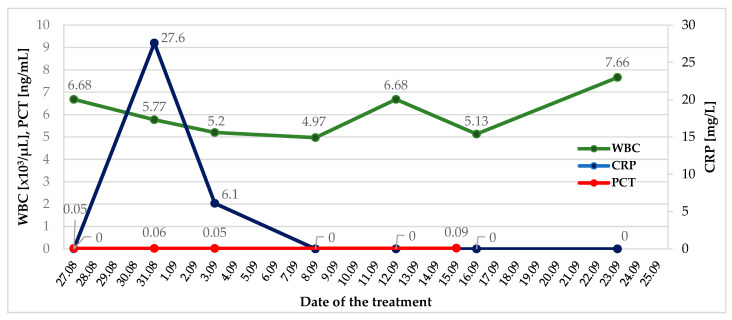
Changes in inflammatory markers during in-hospital observation. WBC—White Blood Cells; CRP—C-Reactive Protein; PCT—Procalcitonin.

However, laboratory tests revealed no significant abnormalities. Inflammatory markers (white blood cells, C-reactive protein, procalcitonin) were within normal ranges, except for a slight increase in C-reactive protein during the perioperative period, likely due to tissue damage (Figure 2).

Transthoracic echocardiography revealed a normal left ventricular ejection fraction of 70%, no valvular abnormalities, and smooth lead contours. Given the relatively recent device replacement procedure and the increased risk of infection with multidrug-resistant Gram-positive cocci, one dose of long half-life lipoglycopeptide antibiotic oritavancin was empirically administered as part of the ORI-4-CIEDi pilot study [10].

**Figure 3:**
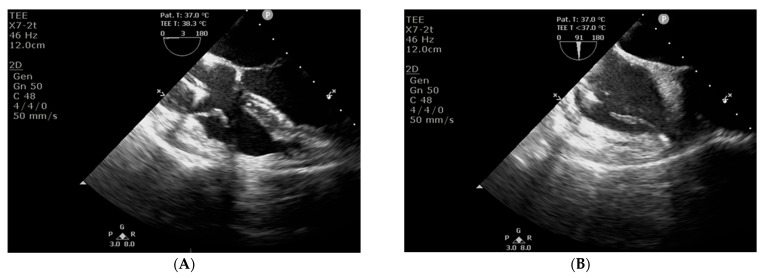
Transoesophageal echocardiography: structures consistent with vegetations on the leads (**A**, long-axis view) and in the right atrium (**B**, short-axis view).

In accordance with the 2019 European Heart Rhythm Association international consensus document on how to prevent, diagnose, and treat cardiac implantable electronic device infections [8], the patient was scheduled for transvenous lead extraction (TLE), which was performed the day after admission. Due to symptomatic bradycardia, the patient was secured with a temporary permanent pacemaker with an active fixation lead. During the TLE procedure (29 August 2025), signs and symptoms of pocket infection were observed, including inflammatory tissue changes and purulent discharge. Routine intraoperative transoesophageal echocardiography (TEE) revealed mobile structures consistent with vegetations on the leads and in the right atrium (Figure 3). CIED-IE was diagnosed. Empirical therapy was expanded to include gentamicin, an antibiotic active against streptococci, enterococci, and Gram-negative bacteria.

**Figure 4:**
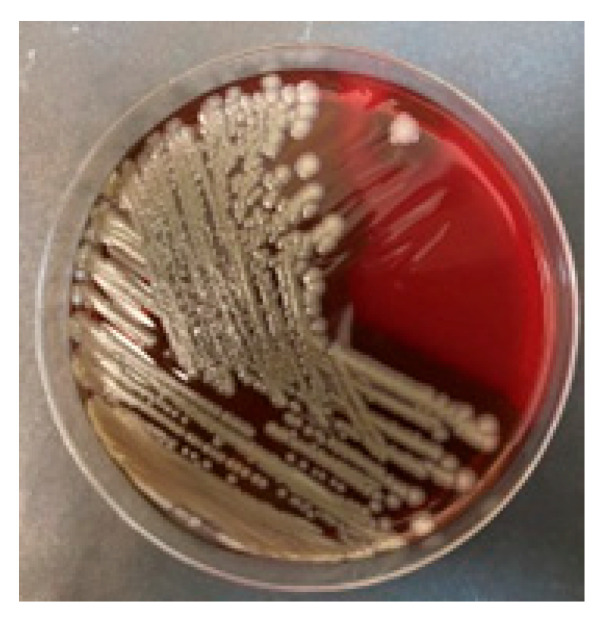
Cultivation of *Bacillus cereus* on blood agar medium.

Surprisingly, despite negative results from multiple blood cultures, Bacillus cereus was cultured from all intraoperative samples collected from the generator pocket and leads (the distal tip, approximately 4 cm long, was collected to protect it from contamination with bacteria from the pocket) (Figure 4). In total, thirteen blood samples were collected, including three before the first dose of antibiotics, three on the day of the TLE procedure, i.e., during the period when postoperative bacteremia could potentially occur, and control samples collected 72 h after the TLE and again after the completion of the next course of antibiotics.

Given the strain’s sensitivity to vancomycin, as confirmed by the antibiogram (Table 1), it was decided to extend the oritavancin treatment by 3 additional fractionated doses at 7-day intervals [11].

**Figure 5:**
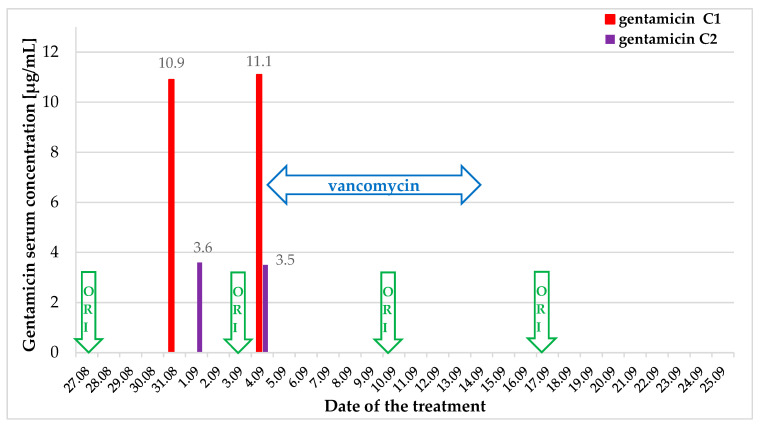
Scheme of antibiotic treatment and gentamicin serum concentration. ORI—oritavancin.

Gentamicin therapy was terminated after 5 days. To eradicate bacteria in the gastrointestinal tract, oral vancomycin (125 mg 4 times a day) was added to the antibiotic regimen (Figure 5, Table 2). No drug-related adverse effects were observed.

**Figure 6:**
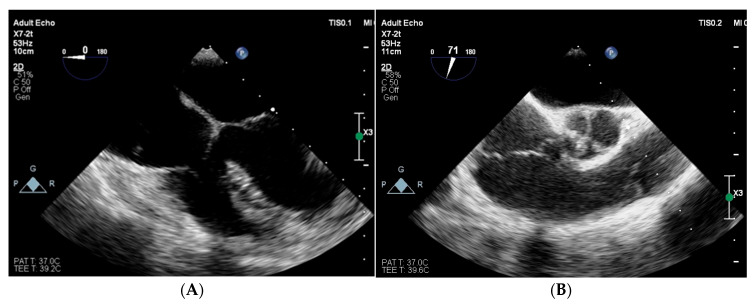
Follow-up transoesophageal echocardiography in long-axis (**A**) and short-axis (**B**) view: previously observed vegetations in the right atrium and the right ventricle have retracted and are no longer visible.

After 4 weeks of targeted antibiotic therapy, the patient showed clinical improvement, including complete wound healing, normalization of inflammatory markers, and absence of vegetations on follow-up TEE (Figure 6).

**Figure 7:**
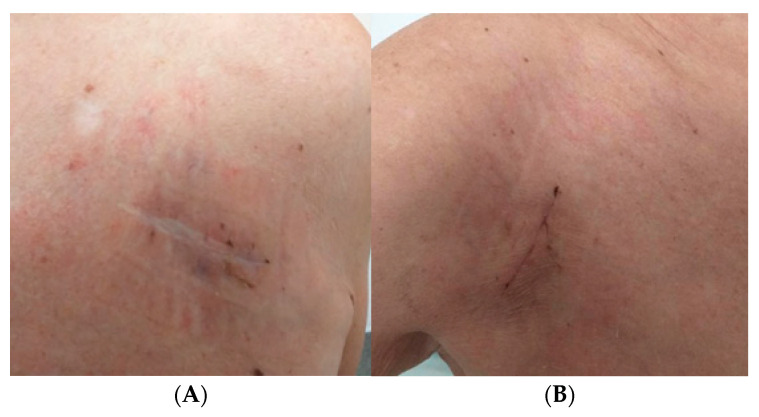
Healed wound after device removal (**A**) and healed wound after device reimplantation on the opposite side (**B**).

Control blood cultures were also negative. On 23 September 2025, a CRT-P device with a left bundle branch area pacing lead was successfully reimplanted in the right subclavian area, and the temporary endocardial lead was removed. The procedure and postoperative period were uneventful (Figure 7). There was no recurrence of infection during the 3-month follow-up.

Notably, the patient received health education and changed her eating habits. The patient diversified her diet, replacing rice dishes with other ingredients, which reduced her habitual constipation. The patient also stopped preparing large quantities of rice and other starchy foods in advance to store until the next day, which will help prevent future food poisoning or recurrence of systemic infection.

## 3. Discussion

The *Bacillus cereus* group (*B. cereus* sensu lato [s. l.]) comprises several species, with *B. cereus* as the most common human pathogen [12,13]. It is a Gram-positive, aerobic, and facultatively anaerobic, spore-forming rod [14]. *B. cereus* is ecologically persistent and can survive in a wide range of environments and temperatures [15]. The presence of spores enables it to survive high temperatures and pasteurization [14]. It is widely distributed in the natural environment, occurring commonly in soil, aquatic environments, vegetables, the gastrointestinal tract of invertebrates, and on human skin [16]. *B. cereus* can contaminate rice and other starchy foods. This bacterium produces two types of toxins: enterotoxin, which causes diarrhea-like food poisoning, and cerulidin, which leads to vomiting-like food poisoning [16]. *B. cereus* is the primary cause of “fried rice syndrome,” a form of food poisoning that arises when cooked rice is left at room temperature for several hours, allowing the bacteria to proliferate. Spores on contaminated rice may survive cooking temperatures too low to destroy their toxins. While cooking reduces spore numbers, it cannot ensure safety. Rice is an excellent medium for bacterial growth, allowing *Bacillus cereus* to thrive under various temperatures, and its spores can remain viable in dried rice for up to 48 weeks. Although B. cereus dies at 45 °C with a water activity of 0.78, most strains are thermotolerant and can withstand mild heat for brief periods [17]. According to the 2018 EFSA report, *Bacillus cereus*, along with its food vehicle (i.e., mixed food), is among the 10 pathogens that cause the highest number of cases, hospitalizations, and deaths in strong-evidence food-borne and waterborne outbreaks, and, compared to data from 2010 to 2017, it indicates increasing trends [18].

Extraintestinal infections caused by *B. cereus* are relatively rare but may occur, especially in immunocompromised patients [19]. These include wound infections, skin/soft-tissue infections, eye infections, endocarditis, postoperative meningitis, urinary tract infections, and liver infections [14,20]. It can also cause pneumonia and sepsis [21,22]. The most common factors predisposing to invasive infections, i.e., endocarditis, include impaired immunity, intravenous drug abuse [23], and the presence of artificial material in the heart [24]. In a French university hospital, Veysseyre et al. [25] observed 57 cases of *B. cereus* infection over a 5-year period and demonstrated that bacteremia was associated with poor prognosis, recurrence, or death. The authors also indicated that in *B. cereus* infections associated with CIED, the device should be removed to avoid recurrence [25].

In a large cohort of Spanish patients with IE, 424 cases of IE associated with CIEDs out of 708 CIEDs (i.e., 59.89%) were identified [26]. In turn, compared to the entire analyzed IE group (*n* = 3996), the prevalence of IE associated with CEIDs was 10.61% [26]. CEID-related infections caused by *B. cereus* are usually isolated in single patients, as indicated by the results of larger studies, or they co-occur with other bacteria as polymicrobial infections [27]. In an observational study of more than 1700 Swiss patients with CEID, device-related infection was reported in 15 patients, of whom only 1 man aged 52 years had a culture-positive *B. cereus* [28]. Also, one patient with CEID-related infection caused by *B. cereus* was identified in another research from Switzerland [29]. In turn, in a Chinese cohort of 145 CEID cases with positive culture results, the majority (almost 97%) were monobacterial infections, and the remaining 5 cases were polybacterial infections, including one case of *B. cereus* with *Staphylococcus epidermidis* [27]. Only a few case reports of CIED-related infections caused by *B. cereus* have been described in the literature [30,31].

According to the European Society of Cardiology guidelines on the management of endocarditis in cases of CIED-related infection, immediate initiation of empirical antibiotic therapy is recommended, covering methicillin-resistant Staphylococci and Gram-negative bacteria, after at least three blood cultures have been obtained [32]. According to established guidelines, the first-line antibiotics used are vancomycin in combination with a third-generation cephalosporin or an aminoglycoside—all administered only parenterally. *B. cereus* is susceptible to chloramphenicol, clindamycin, vancomycin, and erythromycin [33]. *B. cereus* is also considered susceptible to aminoglycosides and fluoroquinolones, as well as newer antimicrobial drugs (linezolid, daptomycin, telavancin) [23], used in the parenteral treatment of CIED-related infections. Only a few studies have shown that *B. cereus* may reveal new resistance to commonly used antibiotics, such as ciprofloxacin, cloxacillin, erythromycin, tetracycline, and streptomycin [34,35]. Some studies have also shown carbapenem-resistant *B. cereus* bacteremia [36,37], and in vitro studies have shown that *B. cereus* may possess a genetically determined metallo-beta-lactamase [38,39]. However, *B. cereus* is usually resistant to penicillin and other β-lactam antibiotics, including cephalosporins [22,33], used in the oral or initial treatment of less severe infections, such as early superficial site infection or isolated pocket infection in an environment with a low prevalence of methicillin-resistant Staphylococci. Therefore, standard empirical treatment would be ineffective.

In the case discussed, only the rapid patient qualification for surgical treatment (TLE) at the reference center enabled identification of the bacterial species and confirmation of the strain’s drug susceptibility without delay. The significant issue was the absence of pathogen growth in blood samples, which hindered the use of rapid microbial species identification methods, such as mass spectrometry, and the performance of antibiograms that require positive blood cultures. This is especially true because samples collected from a fistula or skin erosion over the device, even when available, are insufficient microbiological material for identifying the etiologic agent of CIED-related infection [8]. Furthermore, atypical microorganisms may require more extended incubation periods or specialized growth media, thereby increasing the false-negative rate in microbial samples. In cases of bacterial growth in blood cultures, the applicability of some identification methods, such as polymerase chain reaction (PCR)-based testing (e.g., multiplex PCR), is often limited to typical pathogens and does not allow identification of rare microorganisms or their resistance mechanisms, including *B. cereus*. Similar challenges that hinder identification and effective antibiotic therapy can occur with other rare or opportunistic infections. Therefore, a detailed history is important, along with consideration of changes in the etiology of infections driven by lifestyle, dietary habits, and immune status in our patients.

Notably, in our case, the patient received empirical treatment with the new second-generation semisynthetic lipoglycopeptide antibiotic, oritavancin, as part of a clinical trial ORI-4CIEDi [10]. Oritavancin is an effective, fast-acting antibiotic that acts against Gram-positive cocci by inhibiting cell wall biosynthesis and disrupting bacterial cell membrane integrity [40]. On the other hand, the half-life of oritavancin is 10 to 17 days, which ensures that antibiotic concentrations in serum and deep tissues remain above the minimum inhibitory concentration for several weeks [41]. Thus, it enabled quick and adequate perioperative antibiotic protection and long-term effective treatment of CIED-IE caused by this unusual pathogen. To our knowledge, this case represents the first successful treatment of CIED-IE caused by *B. cereus* using a long-acting lipoglycopeptide antibiotic, administered once weekly.

The present study has some limitations. It is based on a single patient history and demonstrates individual observations; therefore, findings cannot be generalized to incidence, prevalence, or risk in broader populations.

## 4. Conclusions

Infection with rare or opportunistic microorganisms may require extended microbiological diagnostics and non-standard antibiotic therapy; therefore, the medical history should consider risk factors for such infections. In the patient, a diet rich in rice and improper storage of rice dishes, together with habitual constipation, were identified as risk factors for the development of invasive *Bacillus cereus* infection. However, the long half-life lipoglycopeptide antibiotic, oritavancin, administered weekly, was effective in treating CIED-IE.

## Figures and Tables

**Table 1:** Antimicrobial susceptibility of *Bacillus cereus* isolates cultured from intraoperative samples.

Antibiotic	Interpretation
imipenem	S
meropenem	S
erytromycin	S
clindamycin	S
ciprofloxacin	I
levofloxacin	I
vancomycin	S
linezolide	S

S—susceptible; I—susceptible, increased exposure.

**Table 2:** Antibiotic treatment and microbial investigation.

**Day of the Treatment**															
	27 August 2025	28–30 August 2025	31 August 2025	1 September 2025	2 September 2025	3 September 2025	4 September 2025	5–9 September 2025	10 September 2025	11–14 September 2025	15–16 September 2025	17 September 2025	18–20 September 2025	21 September 2025	22–25 September 2025
**Antibiotics**															
Oritavancin (Tenkasi) *	1200 mg					800 mg			800 mg			800 mg			
Gentamicin **			320 mg	280 mg	280 mg	280 mg	280 mg								
Vancomycin ***							250 mg	250 mg	250 mg	250 mg					
**Culture**															
Blood culture				(—)	(—)		(—)							(—)	
Culture of the pacemaker bed				(+)											

*—1200 mg 1×/day on 27 August, then 800 mg 3× every 7 days (i.e., 3 September, 10 September, 17 September), iv; **—in 500 mL 0.9% NaCl 1×/day, iv (12:00); ***—4×/day, po.

## Data Availability

All collected data were presented in the publication.

## Abbreviations

The following abbreviations are used in this manuscript:

CIED	Cardiac implantable electronic device
CIED-IE	Cardiac implantable electronic device-related infective endocarditis
CRT-P	Cardiac resynchronization pacemaker
ORI-4-CIEDi	ORItavancin as a therapeutic regimen for Cardiac Implantable Electronic Devices infections with multidrug-resistant Gram-positive cocci pilot study
TEE	Transoesophageal echocardiography
TLE	Transvenous lead extraction
